# Relapses and Serious Infections in Patients with Neuromyelitis Optica Spectrum Disorder Treated with Rituximab: A Swedish Single-Center Study

**DOI:** 10.3390/jcm13020355

**Published:** 2024-01-08

**Authors:** Olof Carlsson, Dagur Ingi Jonsson, Lou Brundin, Ellen Iacobaeus

**Affiliations:** 1 Department of Clinical Neuroscience, Karolinska Institute, 171 64 Solna, Sweden; olof.carlsson@regionstockholm.se (O.C.); dagur.jonsson@regionstockholm.se (D.I.J.); lou.brundin@ki.se (L.B.); 2 Department of Neurology, Karolinska University Hospital, 171 76 Stockholm, Sweden; 3 Department of Neurophysiology, Karolinska University Hospital, 171 76 Stockholm, Sweden

**Keywords:** neuromyelitis optica spectrum disorder, anti-aquaporin-4 IgG, anti-myelin oligodendrocyte glycoprotein IgG, rituximab, relapse, serious infections

## Abstract

Neuromyelitis optica spectrum disorder (NMOSD) is a rare immune-mediated relapsing-remitting disease of the central nervous system. The usage of rituximab, as relapse-preventive therapy, in NMOSD is common. We performed a single-center retrospective cohort study to assess the risk of relapses and severe infectious events (SIEs) in rituximab-treated NMOSD patients. This study included 24 aquaporin-4 IgG+ (AQP4+), 8 myelin-oligodendrocyte-protein IgG+ (MOG+), and 10 double-seronegative NMOSD patients. Relapses were observed in 50% of all patients during a mean treatment time of 4.0 (range: 0.5–8.25) years. The incidence risk ratio (IRR) of relapse was three times higher in MOG+ compared to AQP4+ patients (IRR: 3.0, 95% confidence interval (CI); 1.2–7.7). SIEs occurred in 40% of all patients during follow-up. AQP4+ patients conferred an increased risk of SIEs compared to MOG+ patients (IRR; 5.3, 95% CI; 1.2–24.3). Incomplete CD19+ B-lymphocyte suppression was not correlated with relapse risk (hazard ratio; 1.9, 95% CI; 0.7–5.2), and there was no correlation between IgG-levels and SIE risk (odds ratio; 2.0, 95% CI; 0.8–4.8). In conclusion, considerable risks of both relapses and SIEs were observed in NMOSD patients exposed to rituximab, which underlines the need for close clinical vigilance of disease activity and infections during treatment.

## 1. Introduction

Neuromyelitis optica spectrum disorder (NMOSD), previously known as neuromyelitis optica or Devic’s disease, is a rare autoimmune disease characterized by inflammation and demyelination in the central nervous system (CNS) [1]. The disease presents with a relapsing–remitting course and mainly affects the spinal cord, optic nerves, and brainstem, with visual loss and paralysis as common clinical manifestations. Untreated, NMOSD often leads to devastating neurological sequelae [2]. Despite being reminiscent of multiple sclerosis (MS), discriminative clinical and radiological criteria for NMOSD have been available since the 1990s [2] and the discovery of IgG antibodies (abs) against the water channel aquaporin-4 (AQP4) has contributed significantly to improve diagnosis and treatment [3].

Around 10–30% of patients fulfilling the 2015 diagnostic criteria for an NMOSD diagnosis [4] are seronegative for AQP4-IgG [5,6] in which a proportion of them rather present with IgG-antibodies against myelin oligodendrocyte glycoprotein (MOG) [7]. Detection of MOG-IgG can be found in patients with different clinical manifestations such as optic neuritis, myelitis, brainstem encephalitis, and acute disseminated encephalomyelitis, in addition to the patients with a diagnosis of AQP4-seronegative-NMOSD [8]. In recent years MOG-IgG-associated disease (MOGAD) has been acknowledged as an inflammatory demyelinating disease of the CNS, with distinct immunological characteristics separate from AQP4+ NMOSD [8]. Another subset of patients that fulfill NMOSD diagnostic criteria contains patients with neither detection of AQP4-IgG nor MOG-IgG, commonly referred to as double-seronegative NMOSD [9]. The knowledge about immunopathogenic disease-driving factors in double-seronegative NMOSD is limited. The disorder seems to be more clinically heterogeneous and often confers diagnostic and treatment challenges [9].

NMOSD is a rare disease, with an estimated worldwide prevalence of 0.7–10/100,000 individuals [10]. A significantly increased incidence rate has been found in Sweden over the recent years and is reported to reach 0.79 per 1,000,000 person-years in 2013 [11]. NMOSD patients face a high risk for severe relapses, often with residual disabilities [2,12]. Disease-modifying therapies (DMTs) to prevent relapses are crucial in managing the disease. Approved DMTs for NMOSD have been lacking until very recently when four monoclonal antibodies, sartralizumab, inebilizumab, eculizumab, and ravulizumab, received approvals for AQP4+ NMOSD on the basis of phase-3 clinical trials [13,14,15,16,17,18]. Common treatment recommendations for NMOSD have previously only included off-label regimens with immunosuppressive agents such as azathioprine (AZA), methotrexate, and mycophenolate mofetil (MMF), with support from real-world studies, case series, and small randomized trials [19,20,21,22,23,24]. Following results from a phase-3 randomized placebo-controlled trial [25] in addition to real-world data [26,27,28], rituximab was recommended as first-line maintenance therapy in AQP4+ NMOSD by the European Federation of Neurological Societies [29] and in the 2015 recommendations of the Neuromyelitis Optica Study Group [30] and later updates [31]. Also in Sweden, rituximab has become the preferred first-choice therapy to treat NMOSD as a consequence of recent supportive data, and probably also because of a growing national experience with rituximab in the treatment of MS [32,33]. Furthermore, rituximab was recommended as first-line DMT for AQP4+ NMOSD in recently updated guidelines from the Swedish Multiple Sclerosis Association [34]. Treatment guidelines for relapse preventive therapies in MOGAD and double-seronegative NMOSD are more limited. The beneficial effect of rituximab to suppress disease activity in MOGAD has been demonstrated, but recent data indicated a reduced effect on clinical attacks compared with AQP4+ NMOSD [35,36].

Safety concerns for rituximab as DMT for MS have occurred due to its association with an increased risk of infections [37]. Similar findings of an increased risk of infections in NMOSD and MOGAD patients treated with rituximab were reported [38], although the knowledge about the extent, severity, and types of infections remains unclear.

We performed a retrospective single-center study on rituximab-treated patients with a diagnosis of NMOSD including AQP4+, MOG+, and double-seronegative patients with the aim to study: (1) the frequency and risk of relapses, (2) the incidence and risk of severe infectious events (SIEs), (3) the correlation between CD19+ B-lymphocyte counts and relapse risk, and (4) the association between serum IgG-levels and SIE risk during long-term follow-up.

## 2. Methods

### 2.1. Study Patients

All adult patients (≥18 years) at the Karolinska University Hospital, Sweden, with a diagnosis of NMOSD according to the 2015 international consensus diagnostic criteria were identified in the Swedish Neuroregistry (SMSreg). Patients treated with rituximab between 1 January 2012 and 31 December 2021 were included. The patients with detection of MOG-IgG had been diagnosed and treated according to a diagnosis of NMOSD. This was a consequence of the study period for which the knowledge of MOGAD, as a separate disease, had not been established. According to the very recent proposal for MOGAD diagnostic criteria [39], some of the current patients categorized as ‘MOG+ NMOSD’ also fulfilled the criteria for relapsing-MOGAD because of diagnostic overlap.

This study was approved by the Swedish Ethical Review Authority (Dnr 2020-03942). All patients are registered in the SMSreg, which signifies that the patients consent to all research based on data from the registry. Further acquisition of informed consent was thereby waived, as commonly applied in Sweden for studies using pseudonymized data from national health registries and medical reports.

### 2.2. Data Collection and Analyses of AQP4-IgG and MOG-IgG Serostatus

Electronic medical charts were reviewed to collect patient data including sex, age at diagnosis, co-morbidities, prior and ongoing immunosuppressive therapy, neurological function at rituximab treatment start according to the Expanded Disability Status Scale (EDSS) [40], relapses, symptoms of neurogenic bladder and/or bowel dysfunction and it’s management, infectious events, death, and cause of death. Similarly, results from serological analyses of AQP4-IgG, MOG-IgG, total IgG-levels, CD19+ B-lymphocyte counts, and neutrophile counts were retrieved from the medical charts. A relapse was defined as the appearance of new neurological symptoms with a duration of at least 24 h and absence of evidence of an infection or fever. A SIE was defined as an infection requiring intravenous (i.v.) antibiotics, hospital admission, or death caused by the infection. The laboratory analyses of AQP4-IgG and MOG-IgG were performed at Wieslab Laboratories, Malmö, Sweden, or (since the year 2014) in-house at the Karolinska University Hospital, Stockholm, Sweden. An immunoblot assay was used until the year 2012, whereafter a cell-based assay was utilized.

### 2.3. Statistical Analyses

Data on patient characteristics and rituximab dosing was compiled using descriptive statistics. Continuous variables were described as mean or medians and interquartile ranges (IQR) or ranges. Categorical data were described as values and proportions. Inter-subgroup differences were analyzed with the Mann–Whitney U test for continuous data and Pearson’s chi-squared test for categorical data. Poisson modeling was applied to calculate the incidence risk ratio (IRR) of relapses and SIEs, respectively. Kaplan–Meier estimators were utilized to assess the time to first relapse and first SIE, respectively, and relative differences between the clinical subgroups were analyzed using Cox proportional hazard regression modeling. The median and IQR of CD19+ B-lymphocytes and IgG were plotted separately to examine potential trends over cumulative doses of rituximab in the clinical subgroups. Logistic regression models were used to assess the association between CD19+ B-lymphocyte count and relapses, as well as the association between SIEs and IgG-levels, EDSS at baseline, and prior immunosuppression, respectively. The significance level was set as *p* < 0.05. Statistical analyses were performed utilizing the software Statistica 13 and Stata MP 17.

## 3. Results

### 3.1. Patient Characteristics

All 42 patients included in this study fulfilled the 2015 criteria for an NMOSD diagnosis [4], patient characteristics are described in Table 1. Based on serostatus, the patients were subgrouped as follows: AQP4+ (*n* = 24; 92% females), MOG+ (*n* = 8, 50% females), and double-seronegative (*n* = 10; 70% females). The mean age at rituximab treatment start was 50, 37, and 41 years for AQP4+, MOG+, and double-seronegative patients, respectively. The median disease duration at rituximab initiation was 14.5 months (IQR: 3–72) for the total study cohort: 48 months (IQR: 2–96) for AQP4+, 16 months (IQR: 2.5–48) for MOG+, and 8 months (IQR: 5–36) for double-seronegative patients. Neurological disability, assessed by EDSS, at the start of rituximab, was comparable between the subgroups with a mean EDSS of 4 (range: 2–7) overall. Six patients, all among the AQP4+ patients, had co-morbid rheumatologic disease including systemic lupus erythematosus (*n* = 4), Sjögren’s syndrome (*n* = 1), and rheumatoid arthritis (*n* = 1). A history of a prior malignant disorder was found in two patients: one AQP4+ patient with breast cancer and one double-seronegative patient with cervix cancer. Immunosuppressive therapy had been prescribed in 12 patients prior to the start of rituximab, mainly AQP4+ patients (*n* = 9), including AZA (*n* = 10), cyclophosphamide (*n* = 3), methotrexate (*n* = 1), interferon-beta-1a (*n* = 1), and fingolimod (*n* = 1).

### 3.2. Rituximab Treatment

Rituximab was prescribed as first-line DMT in 62% of the patients; the dosing regimen is described in Table 2. The induction dose varied between 100 and 2000 mg, the mean maintenance dose was 100–1000 mg, and the mean cumulative dose was 4640 mg (IQR: 1000–8000 mg) in the cohort. Oral prednisolone was administered intermittently concomitant in 18 AQP4+ patients, eight MOG+ patients, and ten double-seronegative patients. Concomitant treatment with AZA was noted in six AQP4+, one MOG+, and two double-seronegative patients. Tocilizumab had been used in parallel with rituximab in two AQP4+, two MOG+, and one double-seronegative patient. The mean follow-up time, defined as the number of years from the initial dose of rituximab to six months after the last dose of rituximab, or to study end, was 4.4 (range: 0.9–7.7) for AQP4+, 2.6 (range: 0.5–4.8) for MOG+, and 4.2 (range: 0.8–8.3) years for double-seronegative patients.

More than a third of all patients (38%) discontinued rituximab treatment during follow up. Within the AQP4+ group, seven (29%) patients discontinued treatment due to the following reasons: new disease activity (of which one patient had anti-rituximab antibodies (*n* = 3), SIEs (*n* = 1), IgG-hypogammaglobulinemia (*n* = 1), emigration (*n* = 1), and old age and long-term stable disease (*n* = 1). Five (63%) MOG+ patients stopped rituximab due to new disease activity, including one that also experienced several infections including one SIE. Four (40%) double-seronegative patients stopped treatment due to insufficient B-cell depletion (*n* = 1), new disease activity (*n* = 1), SIE (*n* = 1), and IgG-hypogammaglobulinemia (*n* = 1). One double-seronegative patient was diagnosed with lymphoma after receiving a total of 1500 mg rituximab. Malignancy was not detected in the AQP4+ and MOG+ groups during follow up.

### 3.3. Relapse Activity

**Figure 1:**
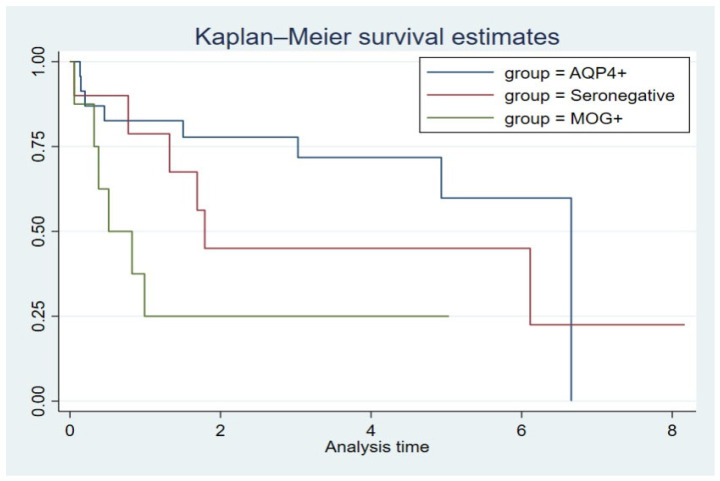
Time to first relapse after the start of rituximab treatment. Y-axis = proportion of relapse-free patients. X-axis = analysis time in years. The vertical decline of the AQP4+ curve at time 6.66 years represents the end of analysis time recording.

Among all patients, 21 (50%) relapsed during follow-up, including nine (37.5%) AQP4+, six (75%) MOG+, and six (60%) double-seronegative patients. The mean ARR was 0.21 (IQR: 0–0.5) in the AQP4+ group, 1.7 (IQR: 0.5–2.6) in the MOG+ group, and 0.48 (0–0.6) in the double-seronegative group. The IRR of having a relapse was three times higher in MOG+ compared to AQP4+ (IRR: 3.0, 95% confidence interval (CI); 1.2–7.7). No significant difference in IRR of having a relapse was found between AQP4+ and double-seronegative patients (IRR: 1.8, 95% CI; 0.7–4.8) or between MOG+ and double-seronegative patients (IRR: 0.6, 95% CI; 0.2–1.8). Kaplan–Meier survival estimates for time to first relapse (Figure 1) demonstrated early relapse activity in the MOG+ and double-seronegative group; 75% of MOG+, and more than half of the double-seronegative patients had relapsed two years after rituximab start. The AQP4+ group exhibited a different pattern with less relapse activity in the first years following treatment start but a persisting risk of relapses after more than six relapse-free years on treatment. A shorter time to first relapse in MOG+ compared to AQP4+ patients was observed (hazard ratio (HR): 3.8, 95% CI; 1.2–11.8), while no differences were found between AQP4+ and double-seronegative patients (HR: 1.7, 95% CI; 0.6–4.6) or between MOG+ and double-seronegative patients (HR: 0.4, 95% CI; 0.1–1.4).

### 3.4. Severe Infectious Events and Malignancy

**Figure 2:**
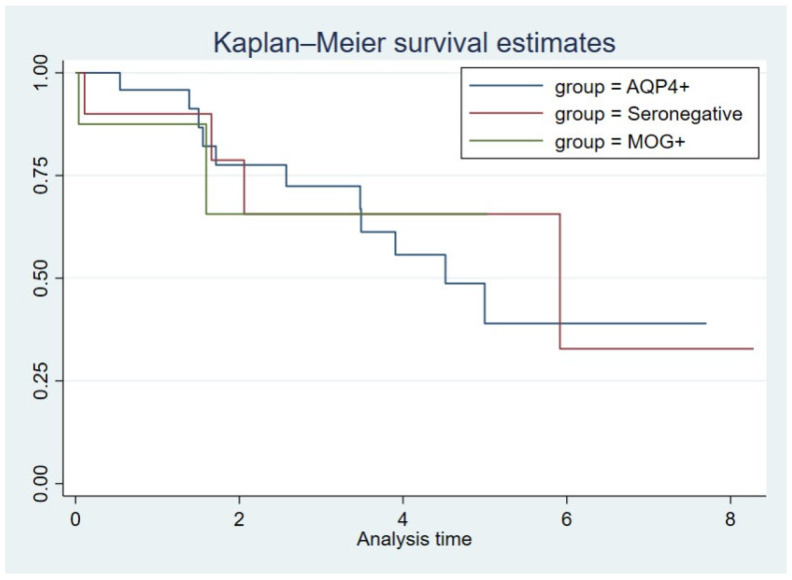
Time to the first severe infectious event after the start of rituximab treatment. Y-axis = proportion of SIE-free patients. X-axis = analysis time in years.

The occurrence of ≥1 SIE was observed in 40% of all patients, including 11 (46%) AQP4+, two (25%) MOG+, and four (40%) double-seronegative patients. The mean annual SIE rate for AQP4+ patients was 0.32 (range: 0–3.3), 0.1 (range: 0–0.6) for MOG+ patients, and 0.18 (range: 0–1.3) for double-seronegative patients. AQP4+ patients conferred an increased risk of SIEs compared to MOG+ (IRR: 5.3, 95% CI; 1.2–24.3). No significant differences between the MOG+ and the double-seronegative group (IRR: 2.4, 95% CI; 0.5–11.3), or between the AQP4+ and the double-seronegative patients (IRR: 0.5, 95% CI; 0.1–1.7), was observed. Kaplan–Meier survival analysis for time to first SIE showed comparable trends between the subgroups, which was confirmed with Cox regression analyses: AQP4+ vs. MOG+ (HR: 1.0, 95% CI; 0.2–2.9), AQP4+ vs. double-seronegative (HR: 1.0, 95% CI; 0.3–2.9), and MOG+ vs. double-seronegative (HR: 1.0, 95% CI; 0.2–6.5) (Figure 2).

Common to all groups, the most frequent types of SIEs were urinary tract infections (UTI), with 50% affected, and upper respiratory infections (50%), pneumonia (27.5%) and bacterial skin infections (27.5%), as described in Table 3. Sepsis occurred in 17.5% of all patients. Three patients (all AQP4+) died during hospitalization for SIE, including one patient with respiratory failure and soft tissue pseudomonas aeruginosa infection, one patient with pneumonia and sepsis with nasopharyngeal culture positive for Streptococcus pneumoniae, and one patient with respiratory failure and chronic decubitus ulcers.

Since UTIs often are associated with neurogenic bladder dysfunction, which is common among NMOSD patients, we collected data on symptoms and management of urinary tract and bowel dysfunction. Five AQP4+, one MOG+, and one double-seronegative patient had urinary catheters (intermittent urinary catheter, indwelling urinary catheter, or suprapubic catheter) during the total (or partial) rituximab treatment time. Among the others, two-thirds of the AQP4+ patients had reported symptoms associated with urinary tract and/or bowel dysfunction, while this was rarer (less than one-third) in MOG+ and double-seronegative patients.

Using logistic regression, adjusted for age at rituximab start, we found no increase in the risk of SIE in patients with moderate/high EDSS (>3) compared to patients with low EDSS (≤3) (HR: 1.6, 95% CI; 0.5–5.8). Furthermore, the frequency of SIEs during rituximab therapy was not correlated with exposure to immunosuppressive therapy prior to the start of rituximab (HR: 3.0, 95% CI; 0.7–12.2).

### 3.5. CD19+ B-Lymphocyte Counts, IgG-Levels, and Neutrophile Counts

**Figure 3:**
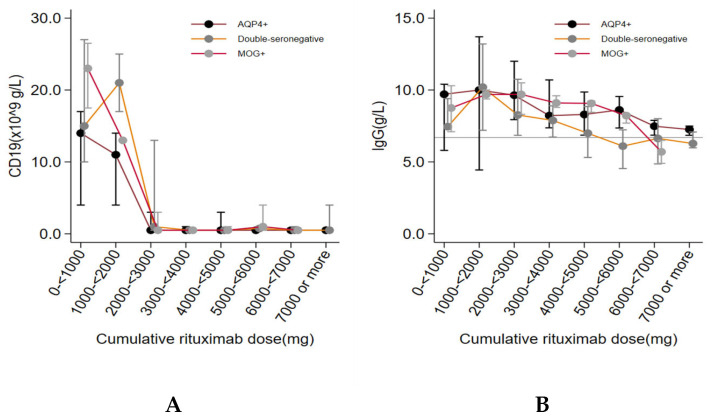
CD19+ B-lymphocytes (**A**) and IgG-levels (**B**) during rituximab treatment.

Complete suppression of peripheral blood levels of CD19+ B-lymphocytes (defined as CD19+ B-lymphocytes < 0.01 × 10^9^ g/L) occurred in most patients when exposed to cumulative doses of 2–3000 mg rituximab (Figure 3A). We found no correlation between relapse and incomplete CD19+ B-lymphocyte suppression (defined as; CD19+ B-cell lymphocyte > 0.01 × 10^9^ g/L) (HR: 1.9, 95% CI; 0.7–5.2).

A non-significant trend of a correlation between decreasing IgG-levels and cumulative doses of rituximab was observed in the total cohort (Figure 3B). The mean level of IgG was below the lower reference limit (<6.7 g/L) at cumulative doses of ≥5000 mg rituximab in double-seronegative patients, and at ≥6000 mg in MOG+ patients, while the majority of AQP4+ patients remained with IgG-levels within the normal reference interval during the study period (Figure 3). A median drop in IgG-levels of 0.35 g/L (95% CI; 0.23–0.47 g/L) per 1000 mg rituximab was detected in the total study group. Logistic regression analyses, adjusted for age, demonstrated no significant correlation between the frequency of SIEs and low IgG-levels (defined as IgG-levels < 6.7 g/L) (odds ratio: 2.0, 95% CI; 0.8–4.8). Only one patient (AQP4+) had persistently decreased levels of neutrophile counts (<1.5 × 10^9^/L) but did not suffer from any SIE during the study period.

## 4. Discussion

This retrospective cohort study observed a substantial relapse- and SIE-burden in rituximab-treated NMOSD patients. Relapses occurred in 50% of all patients, and SIEs were identified in 40% of the patients, during a mean follow-up time of 4.0 years. While MOG+ patients conferred a higher risk of relapses compared to AQP4+ patients, the AQP4+ patients had a five times higher risk of SIEs compared to MOG+ subjects. Rituximab discontinuation was observed in more than a third of the patients (38%). Furthermore, clinical routine monitoring, with an assessment of IgG-levels and CD19+ B-lymphocyte counts, seemed to offer limited value in predicting a relapse and SIE, respectively. Taken together, our results indicated that rituximab failed to control NMOSD disease activity and additionally conferred a high risk of severe infections. Other relapse-preventive therapies, particularly for MOG+ and double-seronegative NMOSD patients, may therefore be considered.

The present finding of 62.5% relapse-free patients in the AQP4+ patients during follow-up is in line with results from a systematic review of rituximab-treated NMOSD patients, which observed 62.9% relapse-free AQP4+ patients during a mean treatment time of 1.0–6.6 years [41].

The efficacy of rituximab therapy in MOG+ and double-seronegative NMOSD patients has been less studied compared to AQP4+ patients. Most evidence of rituximab treatment efficacy in patients with the presence of MOG-IgG abs has currently been provided by studies composed of heterogeneous MOGAD cohorts in contrast to the present study restricted to patients who all fulfilled diagnostic criteria for NMOSD [36]. Our observations are nevertheless in line with previous findings showing a higher occurrence of relapses in MOGAD compared to AQP4+ NMOSD treated with rituximab [35,42]. A recent meta-analysis assessed differences in relapse frequency among MOGAD patients treated with diverse maintenance immunosuppressive therapies, including rituximab, and reported an overall relapse frequency of 62% during a mean treatment period of 1.2 years [43]. The authors concluded that maintenance therapy with i.v. immunoglobin (IVIG) conferred the lowest ARR compared to rituximab, MMF, AZA, and cyclophosphamide, respectively, indicating that IVIG might have been a more effective option as first-line DMT in the current MOG+ patients [43].

It is of value to note that, due to the late introduction of protocols for rituximab treatment for NMOSD at our center, lower annual doses of rituximab had been used, compared to doses administered at other centers, which might negatively have impacted the relapse frequency [26].

The observation of SIEs in 40% of all patients was somewhat unexpected. Lower risks of infections were reported in rituximab-treated MS cohorts, which, however, was anticipated considering the higher frequency of co-morbidities, older age, and difference in disease pathogenic mechanisms, which are attributed to NMOSD [37,44,45]. Previous retrospective studies on AQP4+ and/or MOGAD patients treated with rituximab found SIEs in 8–19% of the patients, which is substantially lower compared to our findings [28,35,38,46]. The differences in follow-up time, age, co-morbidities, and heterogeneous NMOSD cohorts between the studies likely explain the variance in SIE frequency. The current five-fold increased risk of SIEs in AQP4+ patients, compared to the MOG+ group, may likely partly be related to co-morbid rheumatic disease, and associated treatments, which was absent in MOG+ patients. We observed no association between EDSS and risk of SIE, which contrast previous findings [46] but may reflect the relatively low mean EDSS score at treatment start in our patients.

UTIs and upper respiratory infections represented the most common type of infections. Neurogenic bladder and bowel dysfunction are common in NMOSD patients and confer an increased risk for (severe) UTIs. The occurrence of severe UTIs might therefore not entirely been attributed to rituximab treatment. Notable is that five out of seven patients with persisting urinary catheters were AQP4+, which might have contributed to the higher risk of SIEs in this subgroup. We did not have sufficient data regarding the grade of neurogenic bladder and/or bowel dysfunction, and its management, to study their impact on the risk for serious UTIs, which is a limitation of the study. Further studies assessing the association between different types of disabilities, including neurogenic bladder dysfunction and risk of SIEs, are emphasized to increase the knowledge on SIE risk factors in treated and untreated NMOSD patients.

An increasing body of evidence indicates that infections play an important role as triggers for onset and relapses in NMOSD patients [47]. Common colds, otitis media, sinusitis, UTIs, and gastrointestinal infections were reported to precede clinical attacks in NMOSD patients [48]. Postulated mechanisms behind the role of infections in NMOSD disease activity are bystander activation, molecular mimicry, and disease exacerbation by systemic inflammation, involving increased CSF interleukin (IL)-6 levels with promotion of AQP4+ ab secretion from plasma blasts [49]. Our results emphasize the need for high surveillance for infections in rituximab-treated NMOSD patients, in addition to thorough optimization of vaccination strategies. Furthermore, some patients may also require prophylactic antimicrobial therapy.

We observed no apparent correlation between IgG-levels and SIEs. IgG-hypogammaglobulinemia and/or reduced levels of IgG have been reported to be associated with a risk of infections in rituximab-treated patients with rheumatic disease [50], but similar risks in NMOSD patients are less explored. Data from a Korean patient cohort found decreased levels of IgG in 41% of rituximab-treated NMOSD patients, with a mean treatment time of eight years; however, there was no association between risk of infection and hypogammaglobulinemia [46].

The occurrence of neutropenia as a rare side effect has been described in patients with different diseases, including NMOSD [51]. We observed only one AQP4+ patient with persisting neutropenia, which, however, had an absence of SIEs.

The current limited clinical benefit of rituximab despite successful CD19+ B-lymphocyte depletion in combination with a lower risk of SIEs among patients pertaining to the MOG+ group lend further support to the recent recognition of MOGAD as a distinct CNS demyelinating disease [52]. Accordingly, it can be reasoned that other mechanisms are pivotal in the maintenance of autoimmune attacks in MOG+ patients. In addition, the findings of a high frequency of rheumatic diseases in AQP4+, but not in MOG+, nor double-seronegative patients, further argue for diverse mechanisms involved in the dysregulated immune response in the different subgroups of patients fulfilling the present diagnostic criteria for NMOSD [53]. At last, the observation that double-seronegative patients trended towards an increased risk of relapses compared to AQP4+ patients, further highlights the need to stratify NMOSD patients according to serostatus, in clinical practice, to optimize treatment and outcome. Interestingly, a possible beneficial effect of inebilizumab in seronegative NMOSD patients was observed in a post hoc analysis from the N-Momentum trial, which may suggest the need for a more rapid and broader B-cell suppression to prevent relapses in this group [54].

A limitation of the present study is the relatively small sample sizes of subgroups and the intra- and inter-group adjunctive treatment heterogeneity, which may have affected data analysis and interpretation. Further, the relatively late introduction of standard protocols for laboratory monitoring during rituximab treatment in our center has resulted in a few gaps in IgG, and CD19+ B-lymphocyte counts, which might have had an impact on these results. Additionally, the lack of longitudinal EDSS scoring, after rituximab initiation, precluded assessment of EDSS evaluation during therapy, which limited assessment of sustained disability progression in the patients. The long follow-up time in addition to complete data regarding SIEs associated with access to medical charts is a strength of the study. A future prospective study including a larger number of patients and a longitudinal collection of serum immune markers will provide further information regarding risk factors for the development of relapses and SIEs in patients with a diagnosis of NMOSD or MOGAD.

## 5. Conclusions

Although previous studies have suggested rituximab treatment in NMOSD to be effective and safe, our cohort showed a significant burden of relapses and SIEs. The results underline the requisite to find better treatment strategies for NMOSD patients. Furthermore, close clinical surveillance of rituximab-treated NMOSD patients is warranted with allowance for a low threshold for treatment switch. Collecting real-world data is fundamental in understanding the effectiveness and safety of NMOSD and MOGAD treatments and should be systematically applied, especially when applying off-label therapies.

## Figures and Tables

**Table 1:** Baseline characteristics of the study patients.

	Total	AQP4+	MOG+	Double- Seronegative
No. of patients	42	24	8	10
Age at disease onset, mean (range) years	40 (15–72)	42 (21–72)	35 (15–59)	37 (18–71)
Disease duration *, median (IQR) months	15 (3–72)	48 (2–96)	16 (2.5–48)	8 (5–36)
Age *, mean (SD)	45 (16.0)	50 (15.6)	37 (16.7)	41 (13.9)
EDSS * mean (range)	4.0 (2–7)	4.0 (2–7)	3.5 (2–6.5)	4.0 (2–6.5)
Sex (female) *n* (%)	33 (82.5%)	22 (91.7%)	4 (50%)	7 (70%)
Co-morbid rheumatic disease, *n* (%)	6 (14.2%)	6 (25%)	0 (0%)	0 (0%)
Prior malignancy, *n* (%)	2 (4.8%)	1 (4.2%)	0 (0%)	1 (10%)
Prior immunosuppressive therapy	12 (28.6%)	9 (37.5%)	1 (12.5%)	2 (20%)

* At the time when the patient started rituximab treatment.

**Table 2:** Rituximab dosing and relapse frequency.

	Total	AQP4+	MOG+	Double- Seronegative
Induction dose rituximab, range, mg	250–2000	250–2000	500–1000	500–1000
Maintenance dose rituximab, range, mg	100–1000	100–1000	500–1000	500–1000
Cumulative dose rituximab, mean (median), mg	4640 (4100)	4946 (4650)	3281 (3625)	4990 (3850)
Withdrawal of rituximab, *n* (%)	16 (38%)	7 (29%)	5 (63%)	4 (40%)
Follow-up time, mean (min–max) years *	4.0 (0.5–8.3)	4.4 (0.9–7.7)	2.6 (0.5–4.8)	4.2 (0.8–8.3)
Patients (*n*) with a relapse during rituximab treatment	21 (50%)	9 (37.5%)	6 (75%)	6 (60%)
Annual relapse rate; mean (median)	0.55 (0.5)	0.21 (0)	1.70 (1.6)	0.48 (0.3)

* From first rituximab dose to sixth months after the last dose.

**Table 3:** Frequency and characteristics of severe infections.

	Total	AQP4+	MOG+	Double- Seronegative
Patients with ≥SIE *n* (%) *	17/42 (40%)	11/24 (46%)	2/8 (25%)	4/10 (40%)
SIE (mean number)/year	0.24	0.32	0.1	0.18
Death during SIE, *n* (%)	3 (7%)	3 (12.5%)	0 (0%)	0 (0%)
Type of SIE				
Urinary tract infection	21 (50%)	13 (54.2%)	4 (50%)	4 (40%)
Upper respiratory tract infection	21 (50%)	10 (41.7%)	5 (63%)	6 (60%)
Pneumonia	11 (26.2%)	7 (29.2%)	2 (25%)	2 (20%)
Bacterial skin infection	11 (26.2%)	7 (29.2%)	0 (0%)	4 (40%)
Sepsis	7 (16.7%)	5 (20.8%)	0 (0%)	2 (20%)

* During the study period, SIE = severe infectious event.

## Data Availability

Research data supporting the study are available upon request from the author.
